# Dynamics of Random Boolean Networks under Fully Asynchronous Stochastic Update Based on Linear Representation

**DOI:** 10.1371/journal.pone.0066491

**Published:** 2013-06-13

**Authors:** Chao Luo, Xingyuan Wang

**Affiliations:** Faculty of Electronic Information and Electrical Engineering, Dalian University of Technology, Dalian, China; Semmelweis University, Hungary

## Abstract

A novel algebraic approach is proposed to study dynamics of asynchronous random Boolean networks where a random number of nodes can be updated at each time step (ARBNs). In this article, the logical equations of ARBNs are converted into the discrete-time linear representation and dynamical behaviors of systems are investigated. We provide a general formula of network transition matrices of ARBNs as well as a necessary and sufficient algebraic criterion to determine whether a group of given states compose an attractor of length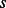 in ARBNs. Consequently, algorithms are achieved to find all of the attractors and basins in ARBNs. Examples are showed to demonstrate the feasibility of the proposed scheme.

## Introduction

The gene regulatory networks (GRNs) as complex systems present diverse dynamical behaviors. In 1969, random Boolean networks (RBNs) were first introduced by Stuart Kauffman to model gene regulatory networks [1]. Each gene is represented by a node in RBNs with two possible states, i.e. the logical 0 and 1. Generally, the “1” value represents the state “on” corresponding to a gene that is being transcribed and the “0” value represents “off” corresponding to a gene that is not being transcribed [2]. A directed edge from one node to another stands for the interaction between genes, the mutual regulation of which is described by an assigned Boolean function. The values of all nodes in a network are updated in parallel and all of them together constitute the state of the whole network. Since dynamics is deterministic and the state space is finite, the states of a particular RBN eventually converge into a series of periodically recurring states, which are called attractors including the fixed points and cycles, starting from an arbitrary initial condition. Attractors and their transient states compose the basins of attraction, which present the dynamical behaviors of systems. RBNs provide a way to formalize large scale complexity in a realistic manner by describing the gene expression with ON/OFF and reflect the statistical features of real living cells by adjusting the parameters of networks [3]. Recent years, along with the research of networks in molecular biology, chemistry, neurobiology, and economy etc [4]–[16], RBNs have been extensively investigated and a wealth of results have been achieved [17]–[28].

As abstract models of physical and biological phenomena, a certain degree of simplification is necessary. Usually, synchrony idealization is adopted as update scheme in the classical RBNs, as Ref. [22], “The simplest class of Boolean network is synchronous, which means that all elements update their activities at the same moment”. All of elements in RBNs evolve according to a global synchronized clock, which is based on a tacit assumption that update scheme would not affect the essential properties of dynamics. However, some doubts have been cast on the validity of the above assumption when more and more evidences showed the dynamic behaviors of systems under asynchronous update presented considerable divergence comparing with the counterparts under synchronous update [29]–[30]. ARBNs was firstly proposed in 1997 by Harvey et al. [31] where only one node was randomly chosen for update at each time step. Since then, many results on ARBNs have been presented and some modified versions of ARBNs were also proposed [32]–[38]. We think, if “synchronous update” is considered as “idealization” [31], Harvey's model as the extreme opposite of synchronous models should be another form of “idealization”. Motivated biologically, since the expression of genes is not an instantaneous process, but the transcription of DNA and the transport of enzymes may take from milliseconds up to a few seconds [36]. So when modeling the above dynamics by means of a discrete form, at each time step, the condition to strictly limit only one node for update is too strong to describe the real situation.

Although RBNs have been much analyzed, but as logical systems, they are difficult to be investigated analytically. Recently, a new matrix theory called the semi-tensor product (STP) was proposed by Cheng et al. [39], whereby logical equations can be represented as a linear discrete-time dynamic equation
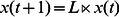
(1)where 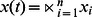 is the semi-tensor product of logical variables. In contrast with usual transition matrix expression, Eq. (1) contains complete information of the logical equations of RBNs, based on which dynamics of systems, such as attractors and basins, can be analyzed. Furthermore, this form can easily be extended to the Boolean control networks. Certain control problems, such as controllability [40], [41], observability [42], and realization [43], etc., can be investigated. So far, the previous studies mainly focus on models under synchronous update. However, ARBNs as nondeterministic systems are considerable different from RBNs. For instance, in Ref. [39], Cheng et al. proposed a method by STP technique to find attractors of RBNs, but, which is based on a fact that RBNs are deterministic systems and attractors are circular. Obviously, this prerequisite can't be satisfied for ARBNs. So, we think it's interesting and meaningful to extend the related research into the field of asynchronous random Boolean networks.

Based on the above discussion, firstly, from the aspect of model selection, most of the previous results on ARBNs are based on the Harvey's model. As mentioned above, we think Boolean networks with a more general asynchronous update schedule are worth investigating. Secondly, from the aspect of methodology selection, despite of the overload of matrix operations, STP technique provides a new perspective to make the analytical analysis of logical dynamics. From the viewpoint of theoretical part, it's a valuable technique to be considered. Therefore, in this article, we focus on dynamics of ARBNs where a random number of nodes can be updated at each time step. Mainly, two parts of work are involved in this article. First of all, we complete an algebraic representation of the studied logical models. At the best of our knowledge, the related result is firstly presented. Network transition matrix takes the essential role for the linear representation of logical dynamics based on STP technique. Actually, in the previous works, Cheng et al. [39] have achieved results of RBNs. Here, we engage in a particular class of ARBNs, from the perspective of update schedule, this special update scheme can involve the case of synchronous update, which allow all of nodes are updated at a certain time step. As a result, a general formula of network transition matrices is achieved, based on which one can calculate all of network transition matrices of a particular ARBN including the counterparts under synchronous update and Harvey's model. Secondly, a necessary and sufficient algebraic criterion is discussed, which determine whether a given group of 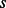 states could compose a loose attractor of length 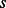 in a specific ARBN. Consequently, a novel approach to detect the attractors and basins of ARBNs is designed, which is completely based on set operations and different from the previous studies.

As we know, to search attractors in RBNs is a NP-hard problem. Recent years, many powerful algorithms [3], [17], [20], [35], [45] have been presented to improve the search efficiency and reduce the time complexity. Yet, it should be noted that our main interest in this article is to provide a complete quantitative method and a novel perspective to analyze the dynamics of ARBNs from the view point of theoretical part. And results would be taken as preparations for the further research, e.g. Boolean control network under asynchronous update.

This article is organized as follows. In the section of Methods, some concepts and properties of STP are introduced. In Results and discussion, the linear representation of ARBNs is discussed, based on which the dynamic properties of ARBNs are studied and algorithms to find attractors and basins of ARBNs are presented. Some examples are shown to illustrate the main results. Finally, a concluding remark is given.

## Methods

In this section, the semi-tensor product of matrices is briefly introduced. Some concepts and properties related to this article are presented.

### Definition 1 ([39])

1. Let 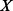 be a row vector of dimension 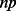, and 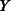 be a column vector of dimension 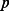. Then we split 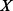 into 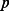 equal-size blocks as 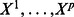, which are 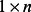 rows. Define the STP, denoted by , as
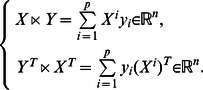

2. Let 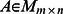 and 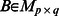. If either 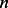 is a factor of 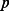, say 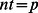 and denote it as 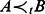, or 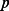 is a factor of 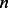, say 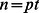 and denote it as 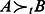, then we define the STP of 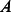 and 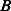, denoted by 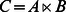, as the following: 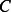 consists of 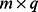 blocks as 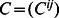 and each block is
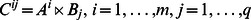
where 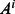 is the 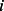-th row of 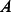 and 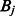 is the 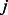-th column of 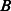.

It's easy to verify that for two column vectors 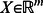 and 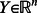, 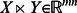.

Note that STP of matrix can be seen as a generalization of the conventional matrix product, so the properties of matrix product, such as distributive rule, associative rule, etc, still hold.

Some notations in this article are defined as follows

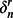
 denotes the 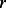-th column of the 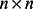 identity matrix 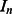 and 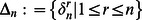, which is the set of all 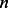 columns of 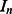. An operation is defined as 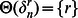, furthermore, when 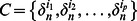, 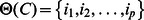.
2. A matrix 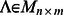 can be called a logical matrix if 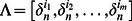, which is briefly denoted by 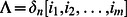. And the set of 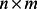 logical matrices is denoted by 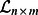.
3. Let matrix 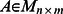, all of elements in 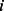-th column of matrix 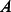 compose a set denoted by 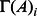, and when a set 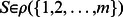, 
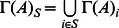
, where 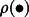 represents the power set.

Next, we define the swap matrix 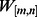, let 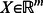 and 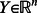 be two column vectors
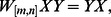
where 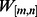 is a 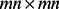 matrix labeled columns by 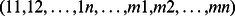 and rows by 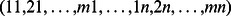, the elements in position 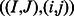 is
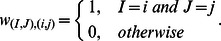


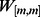
 is briefly denoted by 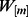.

In order to get the matrix expression of logic, the Boolean values should be denoted as vector forms 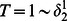 and 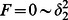. For a logical function 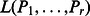 with arguments 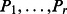, it has been proved in Ref. [39] that 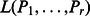 can be represented as 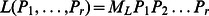, where matrix 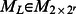 is unique, which is called the structure matrix of logical function 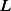.

More details on STP can be found in Ref. [39]. In this article, the matrix products are assumed to be STP and the symbol  is omitted.

## Results and Discussion

### Model

In this section, the algebraic representation of asynchronous random Boolean networks is discussed. Without special note, “asynchrony” in this article implies 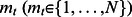 nodes are randomly selected to be updated at time step 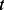. Here, we don't consider the case 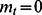 i.e. none of nodes are updated.

ARBNs with 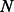 nodes can be described as follows:
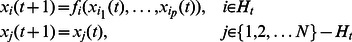
(2)Where 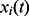 represents the state of node 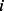 at time 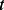, which receives inputs from 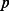 distinct neighbors and 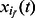 is the 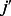 th input. The nodes in ARBNs take values from the set 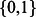 corresponding to two levels of gene expressions. 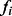 is a Boolean logic from 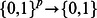, which is assigned to node 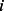. At time 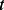, there are 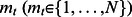 nodes which are selected at random for update, and all of updated elements are included in set 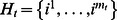, without the loss of generality, we assume that 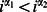 when 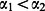. The updated value of selected node 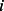 is determined by all of the input values of 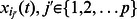 and the logic 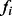. At the same time, the remaining nodes keep the values at time step 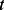.

Firstly, with the structure matrix 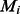 of each Boolean logic 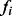, Eq. (2) can be converted into
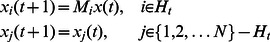
(3)When the in-degree of node 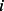 is less than 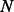, a dummy matrix 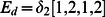 should be applied. It's easy to verify that for any Boolean variable 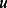, one can get 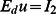. Assume there was no input from node 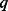 to 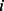, 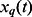 can be substituted by 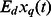.

Multiplying all the equations in (3) as
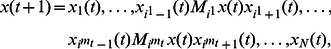
(4)According to Ref [39], when 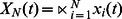 and 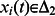, one can obtain 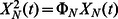, where 
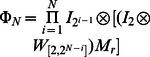
 and 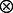 refers to the Kronecker product. Here, 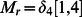, which is power-reducing matrix and it can be verified that 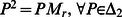.

Then, the following result can be achieved.

**Theorem 1.** Equation (4) can be represented as
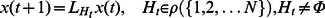
(5)where 
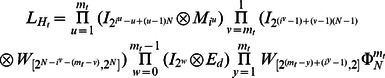
.

### Proof


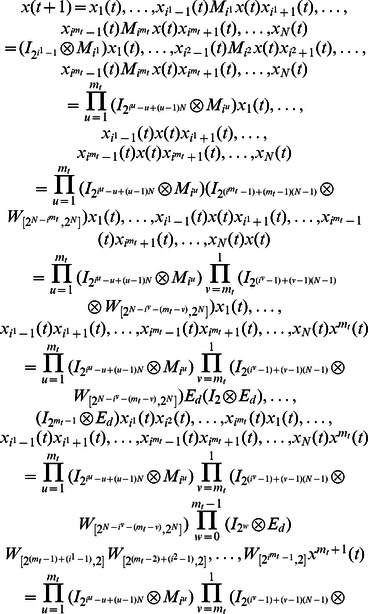
 This completes the proof.

One can prove that the mapping 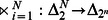 is a bijective mapping. So Eq. (5) can adequately describe the dynamics of Eq. (3) at time 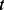, say, 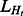 involves all of transition information of system from time 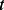 to 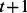 when set 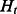 is chosen for update, which is called network transition matrix under update elements 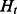.

Whether RBNs or ARBNs, Network transition matrix is an essential concept for linear representation of logical dynamics based on STP technique. As deterministic system, there is only one network transition matrix for a particular RBN. However, multiple matrices exist in ARBNs depending on different asynchronous update scheme. For models discussed in this article, there are total 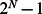 network transition matrices. By means of Theorem 1, when getting the structure matrix 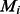 of each Boolean logic 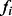 for any specific ARBN, all of network transition matrices could be calculated.

**Example 1.** A logical dynamics is described as
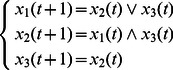
(6)where 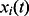 represents the state of node 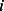 at time 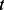, and the symbols “
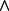
”, “
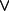
” represent conjunction and disjunction, respectively. By truth tables, one can get 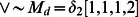 and 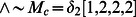.

The algebraic form of Eq. (6) as
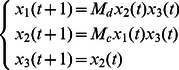
(7)


Case 1: at time 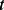, when only node 1 is selected for update,
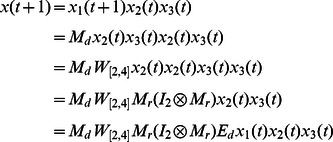



Case 2: at time 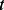, when all of three nodes are selected for update,
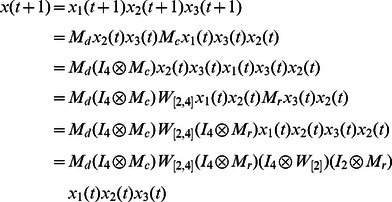
Here, we just show the calculations of network transition matrices under the above two cases. Similarly, all of the network transition matrices can be calculated and results are as follows.
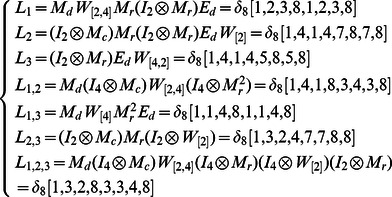



And all of network transition matrices compose a set 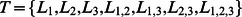.

It should be noted that matrix operations are involved in quantity during the above calculations. Fortunately, the related matrices are typically sparse and many efficient algorithms can be applied to optimize the calculation procedure.

### The asynchronous network transition table

In order to present the algorithm to find the attractors and basins of ARBNs, we first provide the following definition.

**Definition 2.** For a random Boolean network with 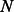 nodes under asynchronous stochastic update, 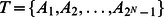 is the set of network transition matrices, where 
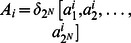
, 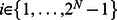. The asynchronous network transition table, briefly called transition table, is defined as
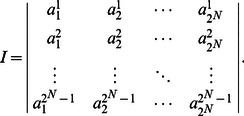
(8)


**Example 2.** Recall Example 1. The transition table of system (7) is as follows
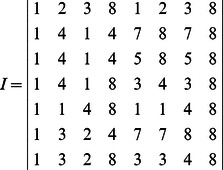
(9)It should be noted that the asynchronous network transition table is specially designed for ARBNs. As mentioned above, more than one network transition matrix exists in ARBNs, which is different from RBNs. Therefore, there is a question: how to organize these network transition matrices can provide an efficient way for applications. For the above consideration, the asynchronous network transition table is presented, by means of which algorithms to find attractors and basins of ARBNs can be achieved completely based on set operations, which are shown later in Algorithms 1 and 2.

### Fixed Points (point attractors)

**Proposition 1.** For a given ARBN with 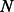 nodes, when 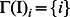, the state 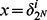 is a fixed point, where 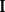 is the asynchronous network transition table.

**Proof.** Assume 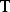 is the set of network transition matrices. When 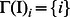, we can obtain 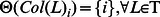, i.e. the 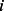-th column of 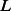 is equal to 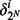, which holds for any network transition matrices in 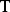. And, it's easily verified that 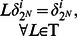, which completes the proof.

Note that 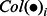 represents the 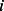-th column of a matrix.

**Figure 1:**
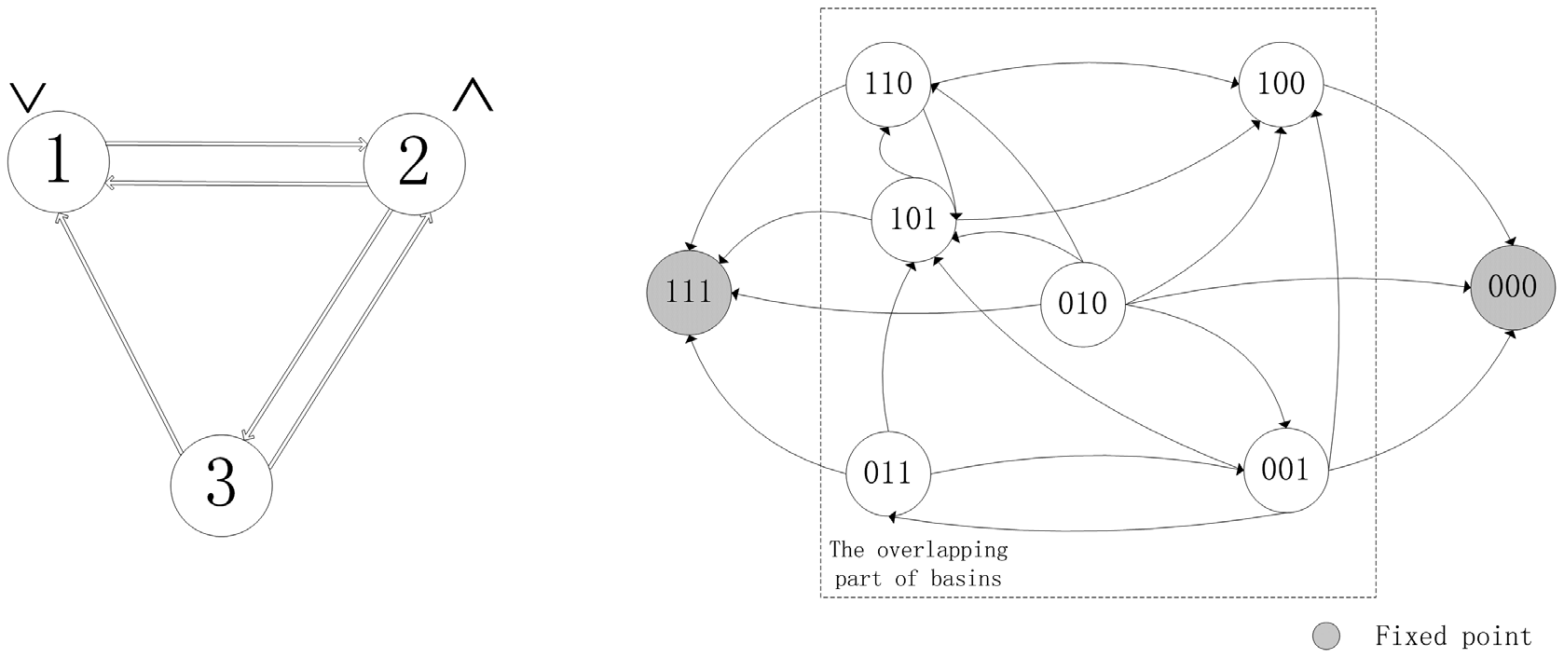
An asynchronous Boolean network with 3 nodes and its state space graph.

**Example 3.** Recall Example 1. We get 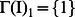 and 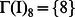. So, two fixed points are 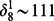 and 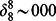, which can be checked in the state space graph of Fig. 1.

### Attractors (Loose attractors)

Fixed points can be seen as point attractors. In the following, we focus on loose attractors in ARBNs.

**Theorem 2.** For a given ARBN with 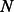 nodes and 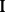 is the asynchronous network transition table, a state set of 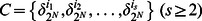 is an attractor of length 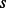, iff
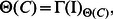
(10)and
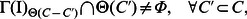
(11)


**Proof.** (Necessity) Assume 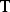 is the set of network transition matrices. When 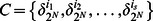 is an attractor of length 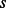, one can obtain 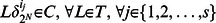, which implies 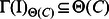. Since all of states in 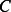 can be reached, that is, for each element 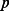 in set 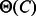, one can find 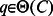 and 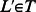 to satisfy 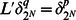, i.e. 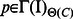, which implies 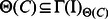. So, one can obtain 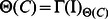 and Eq. (10) holds.

Assume there exists a set 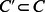, which satisfy 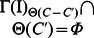. For 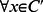 and 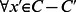, we can't find a path from 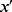 to 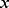, which is contradiction to the definition of attractor. So Eq. (11) holds.

(Sufficiency) Firstly, we should prove that each state in set 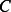 can find a way to reach all of the other states. Assume there exists a state 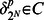 and all of states that 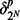 can't reach compose a non-empty set 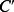. Since 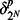
can reach each state in set 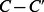, so any states in set 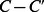 also can't reach states in 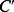, i.e. 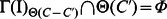, which is a contradiction to Eq. (11). So, each state in 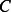 can reach the other states.

Secondly, we should prove each state in set 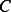 can't reach any states out of set 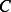.

We assume there exist 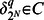 and 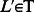 which satisfy 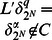. Then 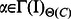 and 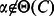, which is a contradiction to Eq. (10).

The proof is complete.

As a consequence, we develop a procedure to find all of attractors of ARBNs based on the above result, that is shown later in Algorithm 1.

### Basins of attractors

**Definition 3.** For ARBNs with 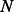 nodes, 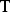 and 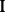 are the set of network transition matrices and transition table, respectively. Let 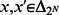 and 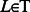, when 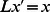, 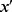 is called the parent state of 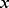. All of parent states of 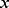 are denoted by the set 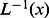. And, for a set 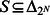, 
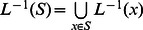
. For simplicity, 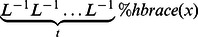 is denoted by 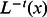.

For nondeterministic systems, a partition can't be found to separate the whole state space of ARBNs into several disjointed sets. As mentioned in Ref. [31], basins of attractors in asynchronous random Boolean networks involve two kinds: the definite basins and possible basins. Then, the overlapping parts of basins of different attractors as common phenomena exist the state space of ARBNs, which mean certain transient states can jump from one basin of attractors into another. More studies related to basins in ARBNs can refer to Refs. [32]–[34].

Generally, by a backstepping manner, one can find the basin of a given attractor 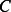. Starting from the state set of attractor 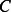, we can determine parent states of each state in 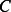, which compose a new set 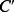. Repeat to find the parent states of each state in 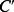 and add them into 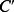 until there aren't any new states involved. The set 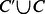 is the basin of attractor 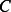. The above procedure is shown in Algorithm 2.

**Example 4.** Recall Examples 2. The attractors of system (7) include the fixed points 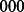 and 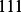. For the fixed point 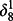, by checking the transition table 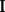 in (9), we can get 
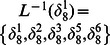
, 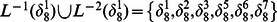. Since 
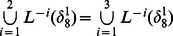
, the basin of the fixed point 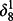 is composed of six states 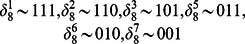. Similarly, for the fixed point 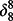, one can get the basin 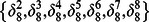. The above results can be verified in Fig. 1.

In the above example, except state 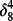 is the definite basin of fixed point 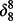, all the rest transient states are the overlapping part between the basins of two fixed points.

### Algorithms for detecting the attractors and basins of ARBNs

Given a specific logical dynamics under asynchronous update, all of the network transition matrices could be calculated based on Theorem 1. Consequently, the asynchronous network transition table can also be achieved. By means of the transition table, we formulate the following algorithms to find attractors and basins of ARBNs.

Algorithm 1: An algorithm for finding attractors in ARBNs with 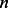 nodes and the asynchronous network transition table 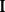.

### Begin: Algorithm 1


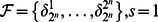
 /* 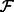 is a feasible state set and 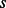 is the length of attractor. */

**for** (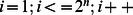) **do** /*find the fixed points in ARBNs */

**if** 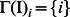 **then** /* 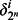 is a fixed point */

  Find the basin of state 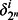; /* the procedure to find basin of a particular attractor refers to Algorithm 2*/

   Remove the state 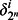 and its basin from set 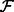;

**end if**

**end for**

**while** Nonempty (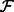) **do**

 

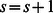


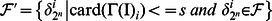
 /* 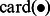 is the number of elements in set. */

  Update 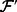 by removing states whose successors aren't in 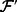.

**while** Nonempty (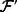) **do**

  Randomly pick up one state 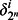 from 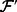 and all reachable states from 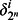 compose a set 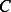.

**if** 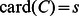 and 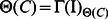 **then** /* 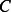 is an attractor of length 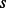 */

   Find the basin of attractor 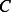 and remove the corresponding states from sets 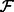 and 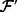.

**else** remove state 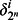 from 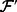


**end if**

**end while**

**end while**

**End: Algorithm 1**

Since, according to the length, the attractors are found successively from small to large, so it isn't necessary to check the condition of Eq. (11) to determine attractors.

Algorithm 2: An algorithm for finding basin of a particular attractor 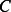 in ARBNs with 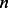 nodes.

### Begin: Algorithm 2


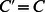
 /* 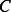 is the set of a particular attractor. */

**while** 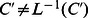 **do**



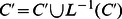



**end while**

**End: Algorithm 2**

In Ref. [39], Cheng's approach to find the attractors of length 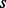 in RBNs needs the computation of 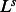, where 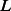 is the network transition matrix. Moreover, Cheng's algorithm is based on a prerequisite that RBNs are deterministic systems and a state on a cycle of length 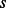 will be back after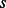 steps. However, this prerequisite can't hold for ARBNs. Therefore, we propose a novel way to detect attractors and basins of ARBNs by means of the transition table.

### Examples

In this section, some examples are presented to illustrate the main results of this article. Firstly, an idealized example is shown as follows, which is a model of phosphorylation/dephosphorylation cycles considered by Gonze et. al [44] and re-investigated in [45].

**Example 5.** A logical dynamics is described as
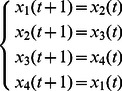
(12)where 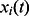 represents the state of node 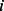 at time 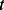. System (12) presents a model of protein–protein interactions. As shown in [45], there are three cycles of length four, one cycle of length two, and two fixed points under synchronous update. Here, we study the attractors and basins of system under asynchronous stochastic update.

The algebraic form of Eq. (12) as
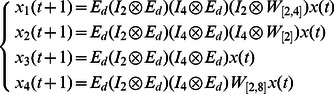
(13)where 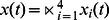.

Then, the structure matrices of logical functions are as follows
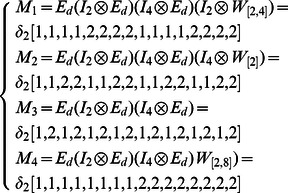
By Theorem 1, all of network transition matrices can be calculated and the asynchronous network transition table is constructed as follows.
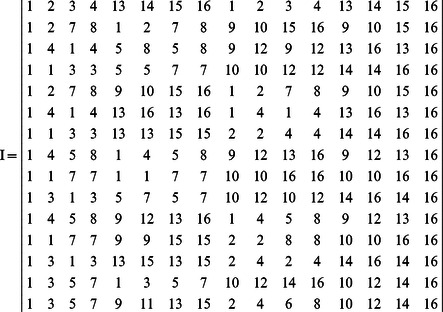
By means of Algorithm 1, since 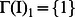 and 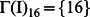 in the transition table 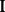, it's easy to get two fixed points 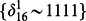 and 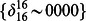. Before finding the attractors of system, we first determine the basins of fixed points by Algorithm 2 to reduce the search scope in state space. For the fixed point 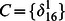, one can get 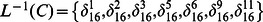 and 
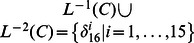
. So far, we can know the basin of the fixed point 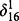 includes all of the states in state space except for the other fixed point 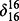. Therefore, we can conclude that there aren't any attractors in this system. Similarly, the basin of the fixed point 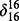 involves all of the states except for 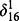. A big overlapping part exists between the basins of two fixed points.

**Figure 2:**
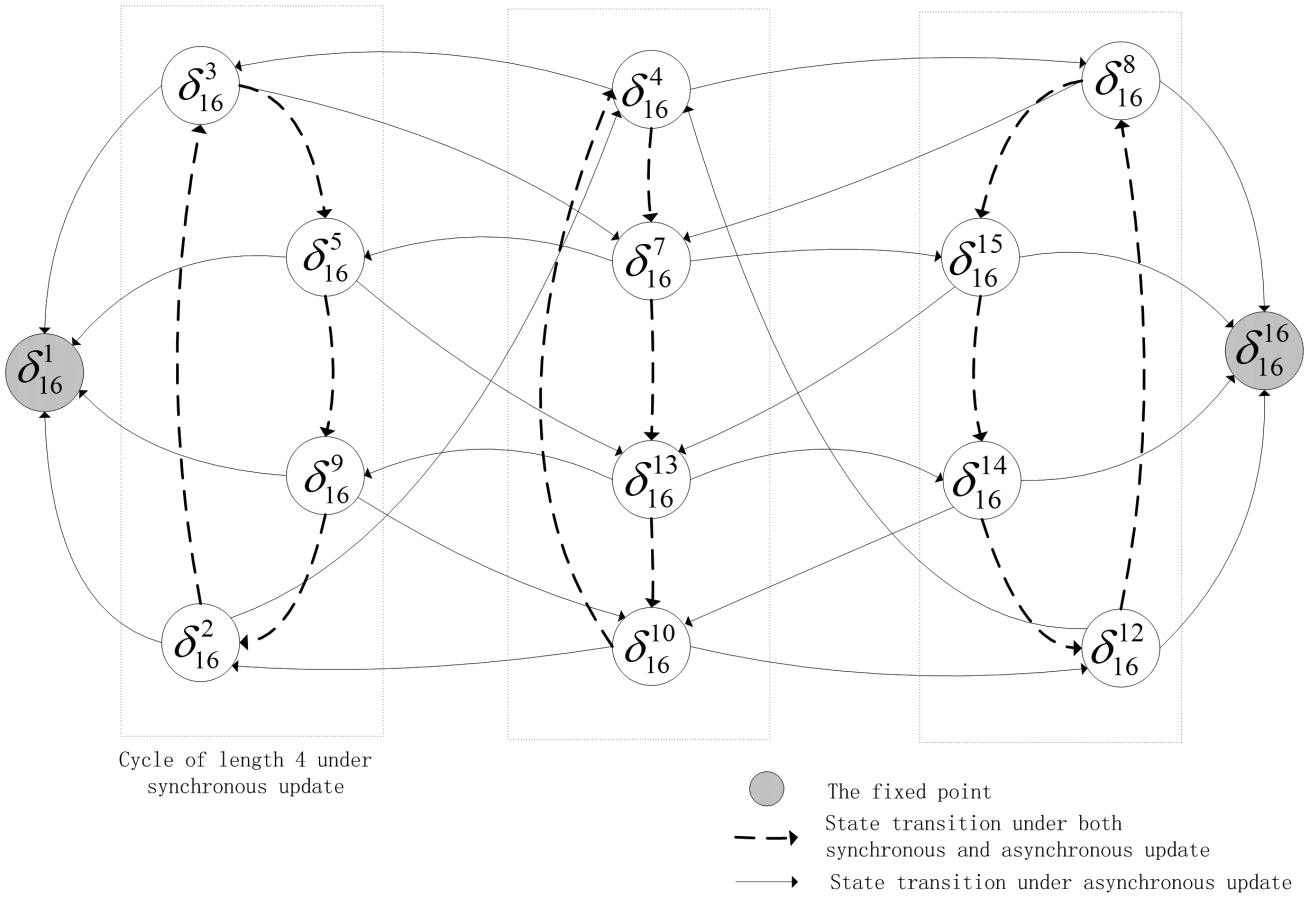
State transfers (Including two fixed points and the former cycles of length four).

**Figure 3:**
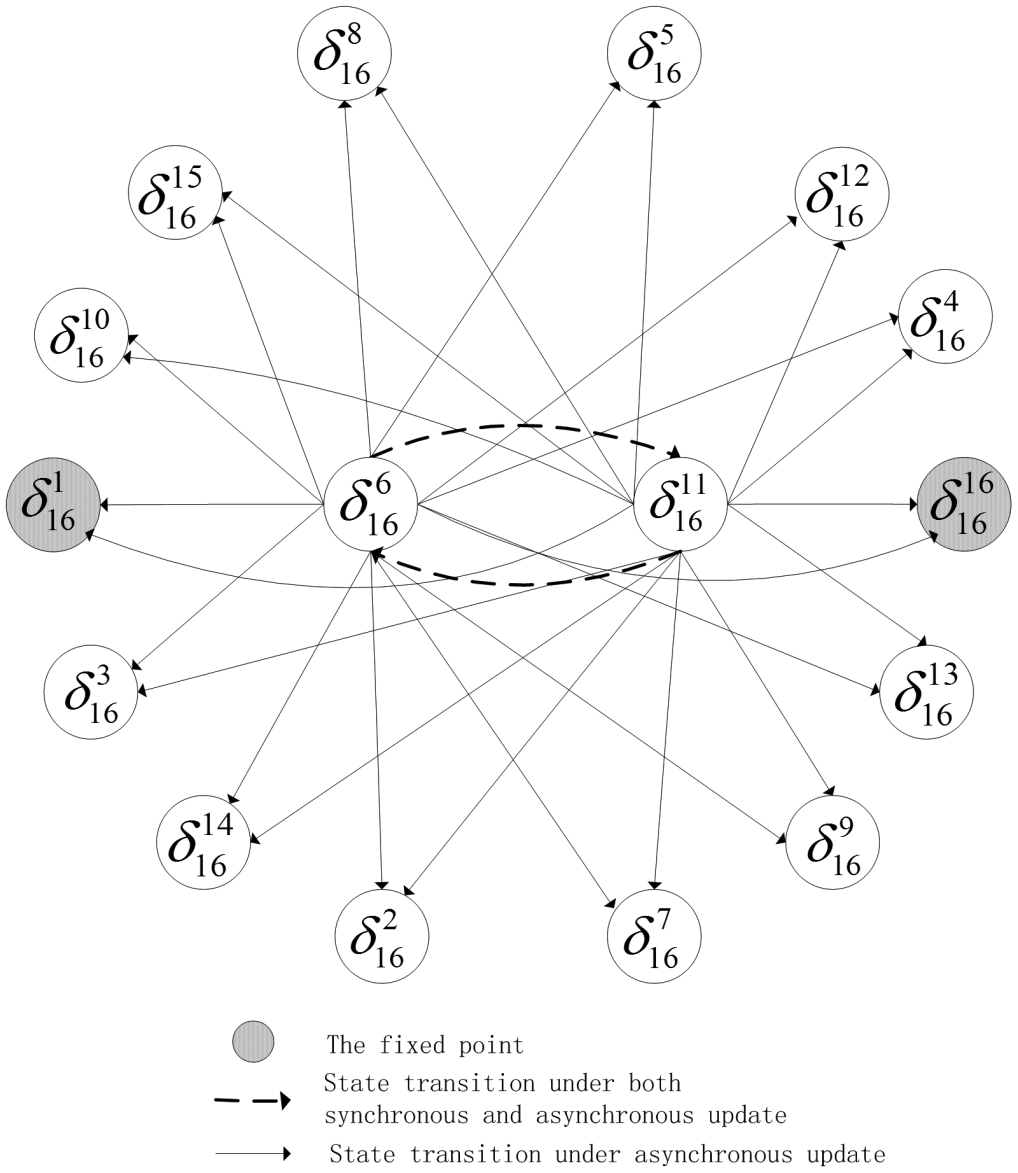
State transfers (The basin of the former cycle of length two).

One can verify, under synchronous update, System (12) has two fixed points as 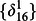 and 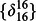, three cycles of length four as 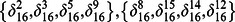 and 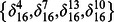, one cycle of length two as 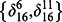. Without ambiguity, we call these cycles under synchronous update as “former cycles”. When system (12) is under asynchronous update, all of former cycles turn into the overlapping part of the basins of two fixed points, say, they can jump from one basin of attractor into another. Furthermore, we also can observe some interesting phenomena. Each state in former cycle 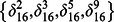 has a direct link pointing to the fixed point 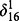, at the same time, all of in-edges of the fixed point 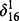 come from this former cycle. The same case occurs between the former cycle 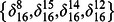 and the fixed point 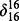. As to the third former cycle 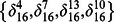, it plays a role of bridge between the rest former cycles of length four, but it hasn't any direct links pointing to either of fixed points. The related state transfers are depicted in Fig. 2. Former cycle 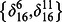 is more special, each state of which has links pointing to all of states in state space except for itself, which is shown in Fig. 3.

Under synchronous update, all of attractors present similar dynamics, e.g. stability, and it's difficult to determine whether there is difference among them. However, some divergences among these attractors can be observed under asynchronous update. Such as the above example in Fig. 2, two former cycles of length four are close to the fixed points, which have greater possibility to flow into the corresponding fixed points. So, we can reasonably consider that the states of the two cycles would be faster into stable states. Yet, the remaining period-4 cycle is in the intermediate position, statistically, whose location determines it would take more time to converge into stability. As to the former period-2 cycle in Fig. 3, to some extent, its characteristic is more unstable under asynchronous update.

**Example 6.** Consider an ARBN with 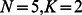, where a random number of nodes can be updated at each time step described as
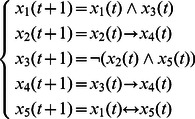
(14)where 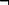, 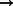 and 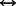 represent the logical functions of negation, implication and equivalence, respectively. One can obtain 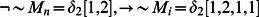 and 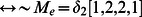.

The algebraic form of Eq. (14) as
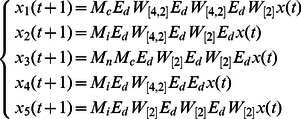
(15)where 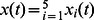.

**Figure 4:**
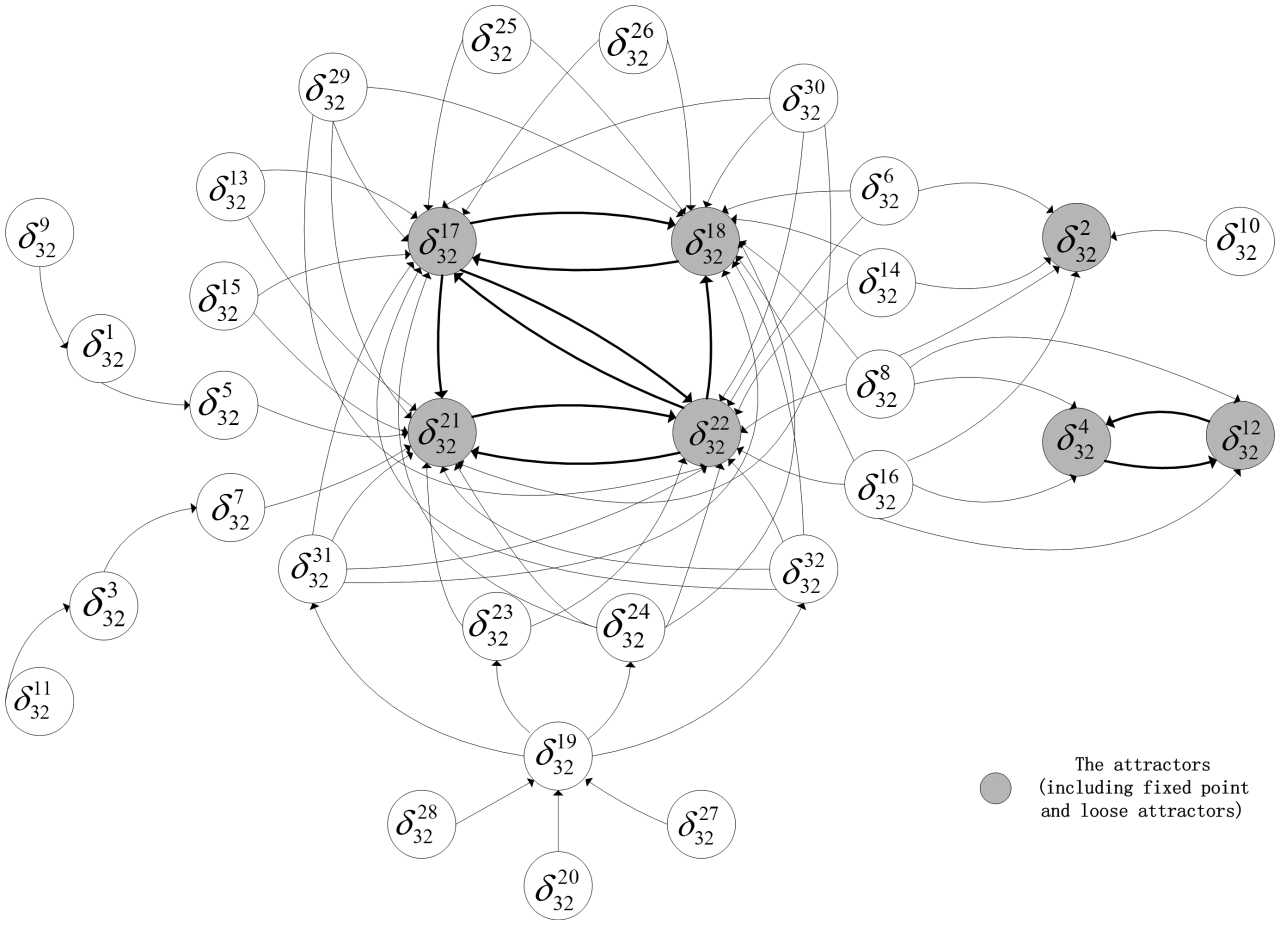
Attractors and basins of system (14).

Then, the structure matrices of logical functions are as follows.



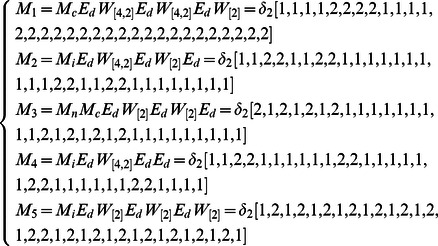
 By Theorem 1, all of network transition networks can be calculated and the asynchronous network transition table is as follows. Construct the feasible set 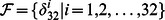. According to Algorithm 1, since 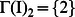, the fixed point 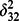 can firstly be found. Consequently, the basin of this fixed point can be detected as 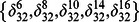 by Algorithm 2. Remove the fixed point and its basin from feasible set 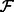. Next, to find the attractors of length 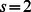, one could obtain 
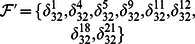
. Since 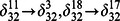 and 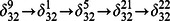, these states should be removed from 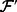 because their successors aren't involved in set 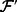. After updating, set 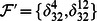. Pick up state 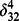 and form the reachable set 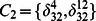. It's easy to check 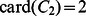 and 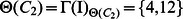, so 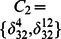 is an attractor of length 2. And, we can find the basin of 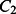 is 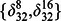. Remove 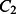 and its basins from 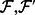 and continue to the above procedure. Similarly, we can find a loose attractor of length 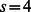 as 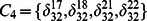 and its basin is 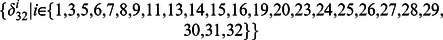. After removing the states 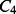 and its basin from 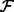, the feasible set 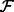 is empty and the procedure is terminated. The state transfer graph is shown in Fig. 4.

In Fig. 4, we can find one fixed point 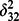, one attractor of length two 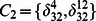 and one attractor of length four 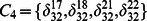. Correspondingly, the basin of each attractor is also depicted. As to fixed point 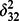, there exist one state 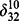 as definite basin and four states 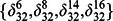 as possible basin. For attractor 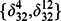, there are total two states 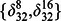 in its basin. Similarly, as to attractor 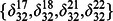, one can find a definite basin 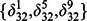 and the rest of transient states in its basin form the possible basin. For simplicity, here, we just show part of state transfers of states in attractors' possible basins.

**Example 7.** A Boolean model of cAMP signaling in Dictyostelium [45]–[47] as follows
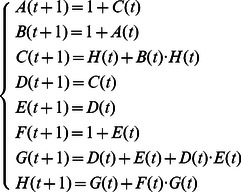
(16)Where the symbols 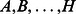 represent PDI, PDE, cAMP(outside the cell), cAR1/3, Erk-2, RegA, ACA and cAMP (inside the cell), respectively. 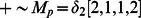 and 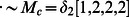. The above biochemical system is known for producing cyclic aggregation signals. In [45], researchers studied the attractors of system under synchronous update and found one fixed point and two cycles of length five. Here, we re-investigate this well-studied system under asynchronous update. Considering the interactions of the elements in the actual model, one element is randomly chosen for update at each time step.

The algebraic form of Eq. (16) is represented as
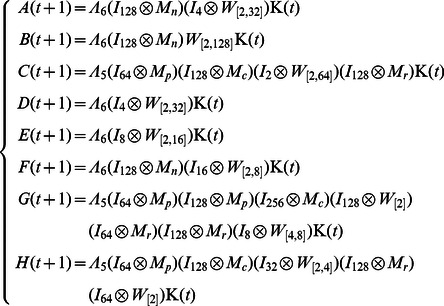
(17)


where 
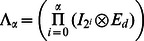
 and 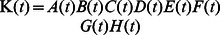.

Based on the above discussions, one can calculate the asynchronous network transition table of system. The detailed computations are skipped and the results are presented as follows.

**Figure 5:**
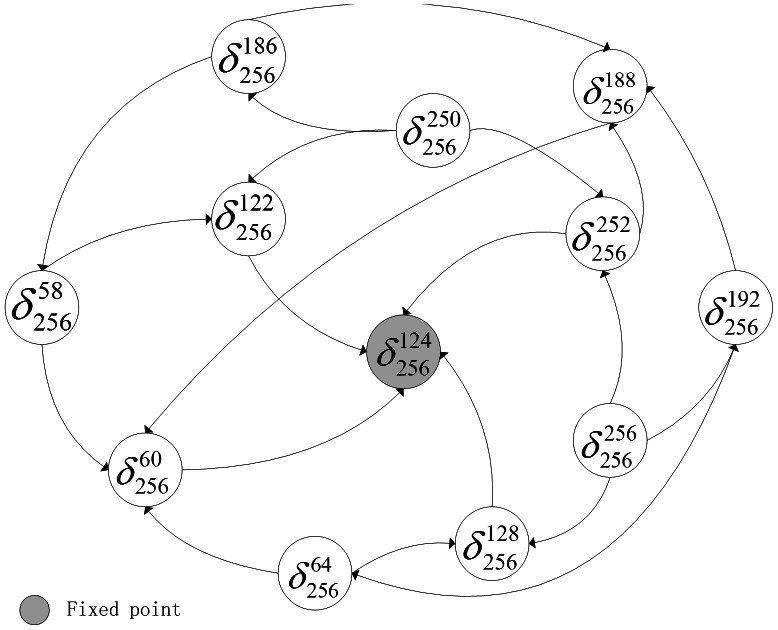
The basin of the fixed point under asynchronous update.

**Figure 6:**
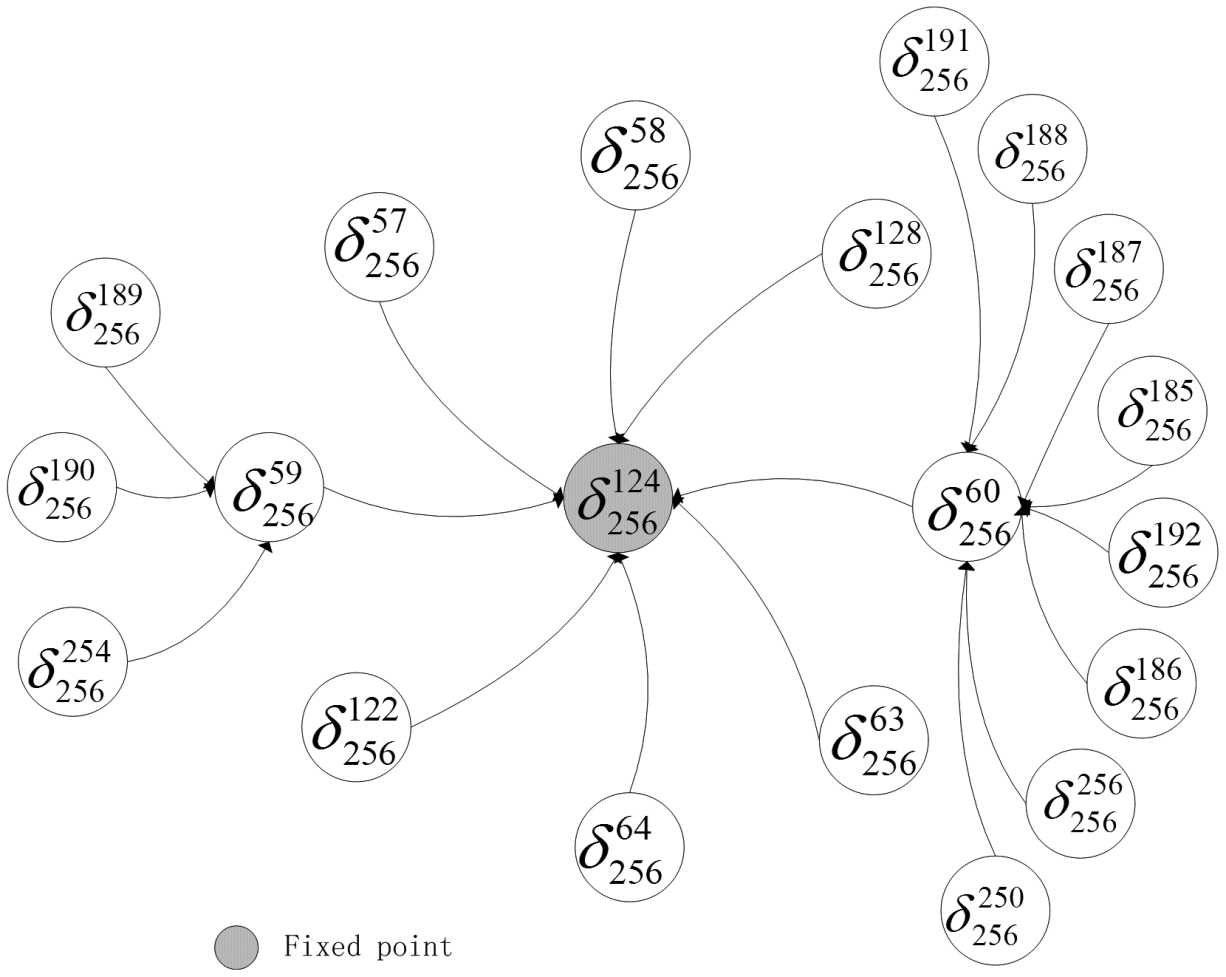
The basin of the fixed point under synchronous update.

It can be verified that only one fixed point 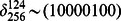 exists in system (16) under asynchronous update, which is the same as the one under synchronous update. Fig. 5 and Fig. 6 show the basins of the fixed point under asynchronous and synchronous update, respectively.

Comparing Fig. 5 with Fig. 6, under asynchronous update, the basin of the fixed point contains fewer states but has more complex interconnections. There are 11 transient states in Fig. 5 and the farthest distance from one transient state to the fixed point is 3 steps, for instance, 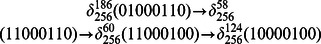. However, in Fig. 6, there are more states directly flowing to the fixed point, furthermore, any states can reach the fixed point though at most 2 steps. There are no attractors in system (16) under asynchronous update, or from another perspective, the remaining states together construct a complicated and big loose attractor.

## Conclusions

In this article, we have presented a new approach to study the dynamics of random Boolean networks under asynchronous stochastic update. By semi-tensor product of matrix, the logic of ARBN is converted into linear representation. A general formula of network transition matrices is presented, by which one can obtain all of network transition matrices of a given logical dynamics whether under synchronous update or under asynchronous update. A necessary and sufficient algebraic criterion has been proved to determine whether a given group of states compose attractor of length 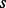 on a particular ARBN. Asynchronous network transition table as a transformed set of network transition matrices is presented. Based on the above results, algorithms to detect the attractors and basins of ARBNs are achieved. Examples are shown to demonstrate the validity of results. As a preparation, results in this article can be applied in the investigation of Boolean control problems under asynchronous update, which would be the work in the future.
